# Supplementary material for: Depletion of ALMS1 affects TGF-β signalling pathway and downstream processes such as cell migration and adhesion capacity
Source: Front Mol Biosci. 2022 Oct 13;9:992313. doi: 10.3389/fmolb.2022.992313 (PMC9621122; doi:10.3389/fmolb.2022.992313)

### **Supplementary Material**:

## Supplementary protocols:

### CRISPR plasmids construction:

Single guides RNAs (sgRNAs) for BJ-5ta model were designing using benchling to generate a deletion of 113pb in the exon 3 of *ALMS1* (ENSG00000116127). The 2 sgRNA (5’- CCTAGTCTGGGATGTATCCA – 3’ and 5’- GAACAGAAGAGATCTCTGTT – 3’) result from benchling were cloned into the plasmid lentiCRISPR v2 (Sanjana et al., 2014) following a modified protocol from that described by Shalem et al (Shalem et al., 2014). LentiCRISPR v2 was a gift from Feng Zhang (Addgene plasmid #52961). Initially, 12.5µL (5µg) of LentiCRISPR V2 were digested with 3µL Esp3I (BsmBI), 3µL FastAP and 6µL 10X FastDigest Buffer from Thermo Fisher. Final volume was set at 60µL. Plasmid digestion was checked by migration in an agarose gel at 1%.

sgRNAs were phosphorylated and annealed with its correspondent oligo mixing as follow: 1µL sgRNA (100µM), 1µL of its complement oligo, 2µL of 5X T4 Buffer (Canvax, Cordoba, Spain), 0.5 T4 PNK (NEB, Ipswich, EE.UU) and 5.5 µL distilled H_2_0 (H_2_Od). Mix reaction was incubated at 37ºC for 30min and 95ºC for 5min. The samples were then left to stand at room temperature for 2 hours and diluted at 1:50, 1:100 and 1:200 ratios.

The unpurified digested plasmid was ligated with the diluted sgRNAs as follows: 0.5µL digested LentiCRISPR v2, 2µL diluted sgRNA, 1µL T4 DNA Ligase (Canvax, Cordoba, Spain), 4.5µL H_2_Od. The mix was incubated at 37ºC by 30min.

Finally, Stable Competent E.coli (NEB, Ipswich, EE.UU) were transformed by thermal shock according to the manufactured protocol and incubated at 30ºC overnight. After that, construct validation was carried out using forward primer for U6 promotor (5’- GACTATCATATGCTTACCGT -3’) and reverse sgRNA.

### Plasmids Transfection and Lentivirus production:

293T were seeded in a concentration of 3x10^5^ into a 6-well plate and incubated overnight. Then, 293T cells were co-transfected with each CRISPR construct and the plasmids psPAX2 and pCMV-VSV-G (Stewart et al., 2003) using lipofectamine 3000 (Thermo Fisher, Waltham, EE. UU). pCMV-VSV-G was a gift from Bob Weinberg (Addgene plasmid #8454) and psPAX2 was a gift from Didier Trono (Addgene plasmid #12260)

Initially, 7.5µL of lipofectamine 3000 were diluted in 125µL of Opti-MEM medium (Thermo Fisher, Waltham, EE. UU) by duplicate. Then a total amount of 5µg of ADN (1.6µL for each plasmid) were diluted at 250µL Opti-MEM. After that, diluted DNA and diluted lipofectamine were mixed in a 1:1 ratio, centrifugated at 1200rpm and incubated for 20min at room temperature. Finally, the 250μL of mix were applied to the wells containing the cells.

The cells were incubated with the mixture for 24 hours. Then, the mixture was removed, and the cell was cultured for a further 72 hours for lentivirus production.

### Lentiviral transduction:

After 72 hours of culturing 293T cells transfected for lentivirus production, the culture medium was collected in 15 mL tubes and filtered through a 45µm filter (Sartorious AG, Göttingen, Germany). In addition, a solution of complete DMEM medium without P/S with polybrene was prepared at a dilution of 1:1000 (8µg/ml).

Then, 300µL lentiviral preparation were mixed with 200µL complete DMEM medium with polybrene for each well. An additional 1 ml of DMEM with polybrene was added and the plates were centrifuged at 800g for 30 minutes. This step was repeated one more time. After the second centrifugation the mix with lentivirus was added again and the plates were incubated for 24 hours. Finally, mix was removed, and cells were allowed to settle for 48 hours in DMEM medium. They were then selected with puromycin (1ng/mL) for 5 days.

### Edition validation:

After puromycin selection, clonal isolation, and expansion we checked the lack of *ALMS1* gene by qPCR in StepOnePlus (Thermo Fisher, Waltham, EE. UU). Finally, we found that these cells had the expected exon 3 deletion by PCR with resolution of samples on a 2% agarose gel and followed by sanger sequencing.

### Bibliography:

Sanjana, N. E., Shalem, O., and Zhang, F. (2014). Improved vectors and genome-wide libraries for CRISPR screening. *Nat. Methods* 11, 783–784. doi: 10.1038/nmeth.3047.

Shalem, O., Sanjana, N. E., Hartenian, E., Shi, X., Scott, D. A., Mikkelson, T., et al. (2014). Genome-scale CRISPR-Cas9 knockout screening in human cells. *Science* 343, 84–87. doi: 10.1126/science.1247005.

Stewart, S. A., Dykxhoorn, D. M., Palliser, D., Mizuno, H., Yu, E. Y., An, D. S., et al. (2003). Lentivirus-delivered stable gene silencing by RNAi in primary cells. *RNA* 9, 493–501. doi: 10.1261/rna.2192803.

## Supplementary Tables:

**Supplementary Table S1.** sgRNAs used to generate knockout models in HeLa and BJ-5ta cells.

| **ID** | **Cell line** | **Exon** | **Forward primer (5' - 3')** | **Reverse primer (5' - 3')** | **Strand** | **PAM** | **On-target Score** | **Off-target Score** |
| --- | --- | --- | --- | --- | --- | --- | --- | --- |
| gRNA 1 HeLa | HeLa | 1 | GCCCCCGCAGCACCGCTACT | AGTAGCGGTGCTGCGGGGGC | + | CGG | 51,4 | 89,3 |
| gRNA 2 HeLa | HeLa | 1 | TCCGCTCGCCCTCCGAGTAG | CTACTCGGAGGGCGAGCGGA | - | CGG | 56 | 91 |
| gRNA 3 HeLa | HeLa | 3 | TTCTCACATTAAACCTAGTC | GACTAGGTTTAATGTGAGAA | - | TGG | 29,1 | 76,7 |
| gRNA 1 BJ | hTERT-BJ-5ta | 3 | CACCGCCTAGTCTGGGATGTATCCA | aaacTGGATACATCCCAGACTAGGCC | - | AGG | 56,2 | 77,1 |
| gRNA 2 BJ | hTERT-BJ-5ta | 3 | CACCGAACAGAAGAGATCTCTGTT | aaacAACAGAGATCTCTTCTGTTC | - | GGG | 63,3 | 58,1 |

**Supplementary Table S3.** Primers used to study the expression of EMT markers and for genotyping the knockout models.

| Gene symbol | Forward primer | Reverse primer |
| --- | --- | --- |
| *POSTN* | CACTCTTTGCTCCCACCAAT | GCCACTTTGTCTCCCATGAT |
| *CDH2* | CCTCCAGAGTTTACTGCCATGAC | GTAGGATCTCCGCCACTGATTC |
| *ACTA2* | CTATGCCTCTGGACGCACAACT | CAGATCCAGACGCATGATGGCA |
| *SNAI1* | GCTGCAGGACTCTAATCCAGAGTT | GACAGAGTCCCAGATGAGCATTG |
| *TWIST1* | GCCAGGTACATCGACTTCCTCT | TCCATCCTCCAGACCGAGAAGG |
| *EDIL3* | GCGAATGGAACTTCTTGGCTGTG | GAGCGTTCTGAAGATGCTGGAG |
| *VIM* | AGGCAAAGCAGGAGTCCACTGA | ATCTGGCGTTCCAGGGACTCAT |
| *DSP* | TGACAGACCGCTGGCAAAGGAT | GGCGTTTAGCATCATAGAGCCAC |
| *ALMS1* (Exon1) | GCACTGCGCCTAAGCTG | gttgggggtggaggctg |
| *ALMS1* (Exon3) | cagttaatgacttagcatgttttcct | ctttcccctttttgagttaagga |
| *ALMS1* (Exon 1 -3) | GAGCGAGACACCAACATGGA | ATTGCGTCAATATGCCCTCCT |

## **Supplementary** Figures:


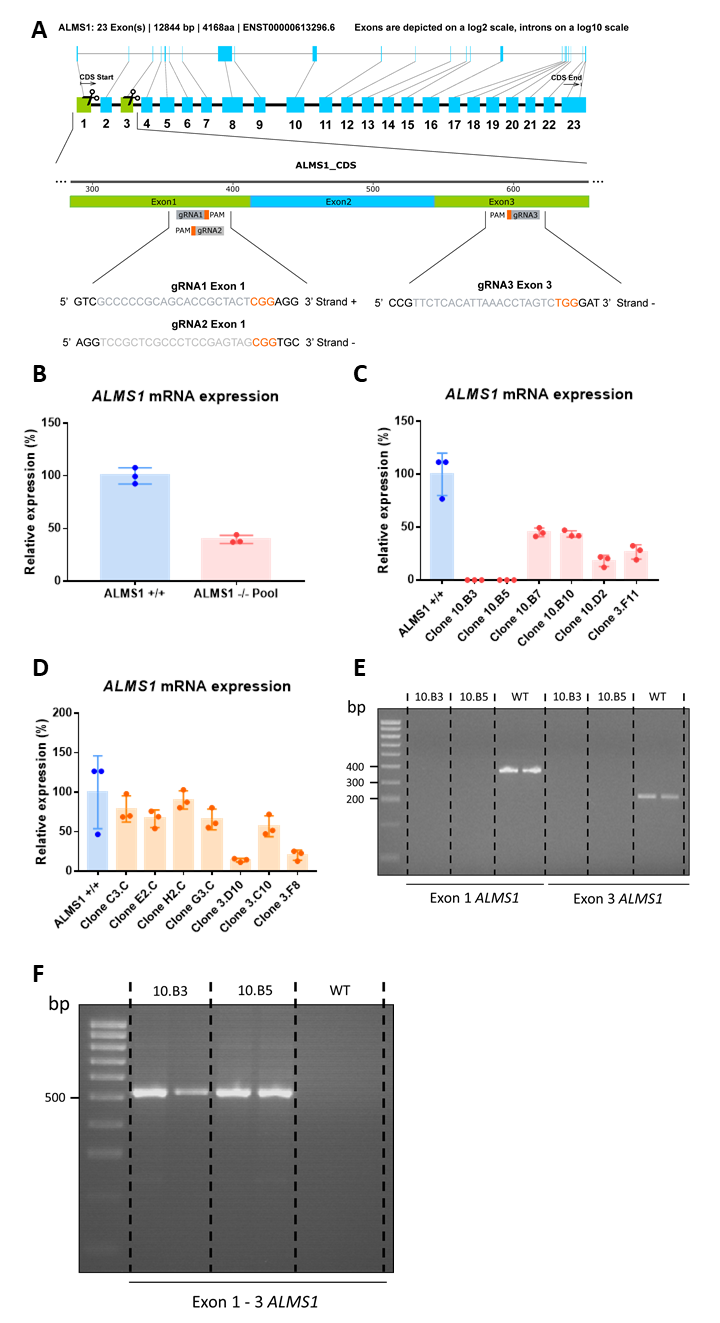


**Supplementary Figure S1.** Characterisation of *ALMS1* -/- cell line HeLa. **(A)** Design of the model. **(B)** Relative gene expression of *ALMS1 +/+* and polyclonal cell line for *ALMS1 -/-* genotype. **(C)** Relative gene expression of *ALMS1* +/+ and a first batch of clones for *ALMS1 -/-* genotype. **(D)** Relative gene expression of *ALMS1* +/+ and second batch of clones for *ALMS1* -/- genotype. **(E)** Strategy for amplification of exons 1 and 3 from genomic DNA in two clones of the *ALMS1* -/- (KO) line and the *ALMS1* +/+ control (WT). **(F)** Amplification strategy of the resulting sequence between exon 1 and 3 after removal of the sequencing between the 2 outermost gRNAs.


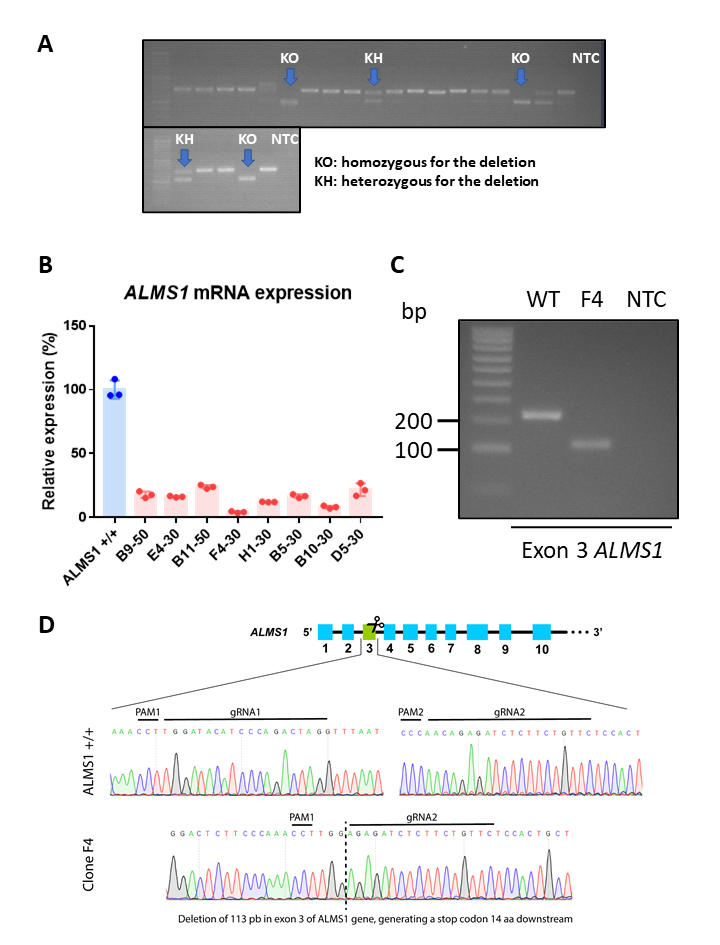


**Supplementary Figure S2.** Characterisation of *ALMS1* -/- cell line BJ-5ta. **(A)** Clone screening in agarose gel. **(B)** Relative gene expression of *ALMS1* +/+ and a batch of clones for *ALMS1* -/- genotype. **(C)** Agarose gel (2%) showing the exon 3 amplicon of the control line (*ALMS1* +/+) and the F4 clone (*ALMS1* -/-) which showed the lowest gene expression. **(D)** Characterisation of the F4 clone mutation by sanger sequencing.

### BJ-5ta western-blots:

Replicate 1 of the WB for the measurement of p-SMAD3


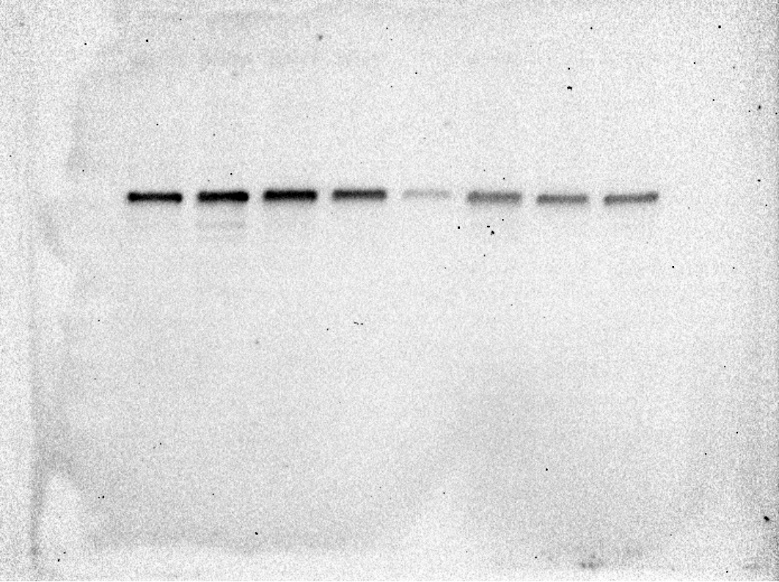


Replicate 2 of the WB for the measurement of p-SMAD3


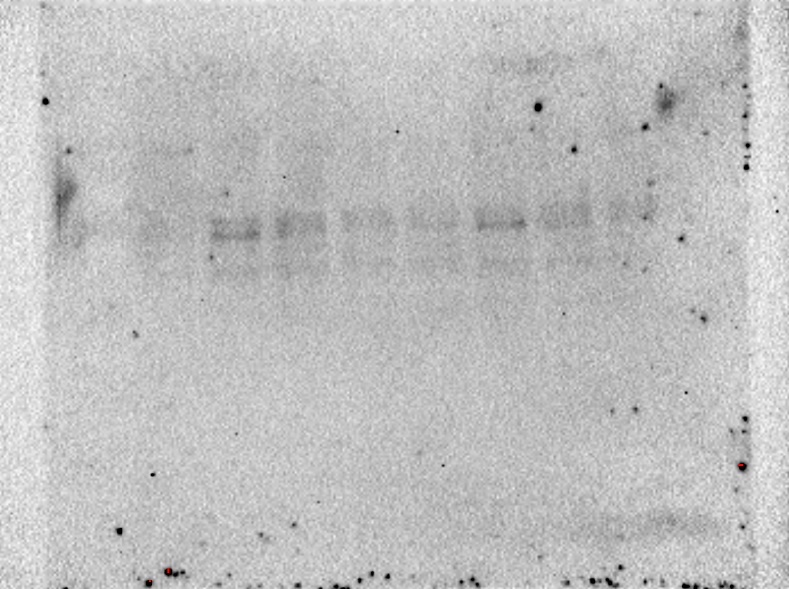


**p-SMAD3**

Replicate 3 of the WB for the measurement of p-SMAD3


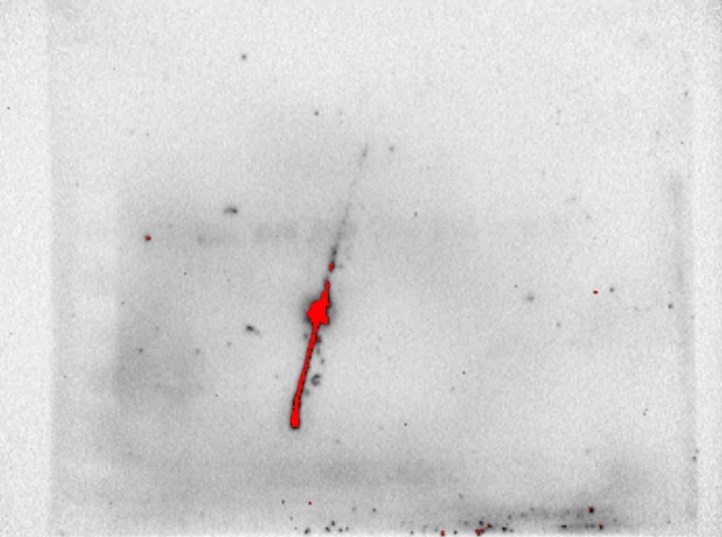


**p-SMAD3**

Replicate used for quantification in p-SMAD3 with independent clones without stimulation (TGF-β1 0 min). The first 4 wells are independent clones of the WT genotype and the next 4 wells are clones B9, B5, B10 and H1 respectively.


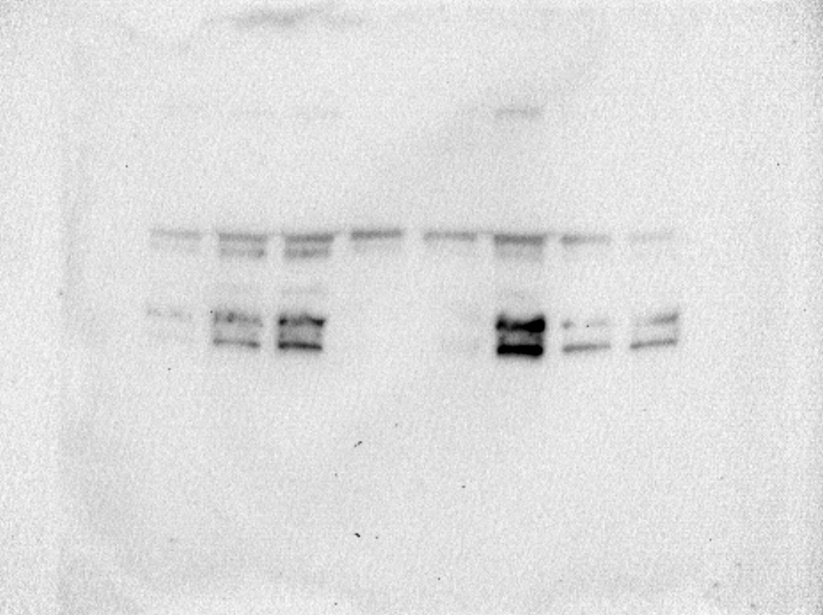


**p-SMAD3**

Replicate used for quantification in p-SMAD3 with independent clones with stimulation (TGF-β1 30 min). The first 4 wells are independent clones of the WT genotype, and the next 4 wells are clones B9, B5, B10 and H1 respectively.


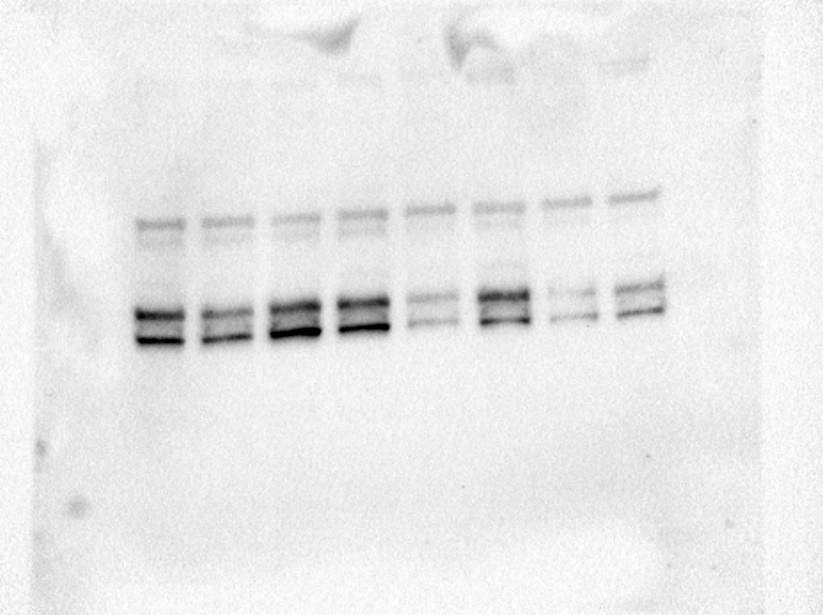


**p-SMAD3**

Replicate 1 of the WB for the measurement of SMAD3


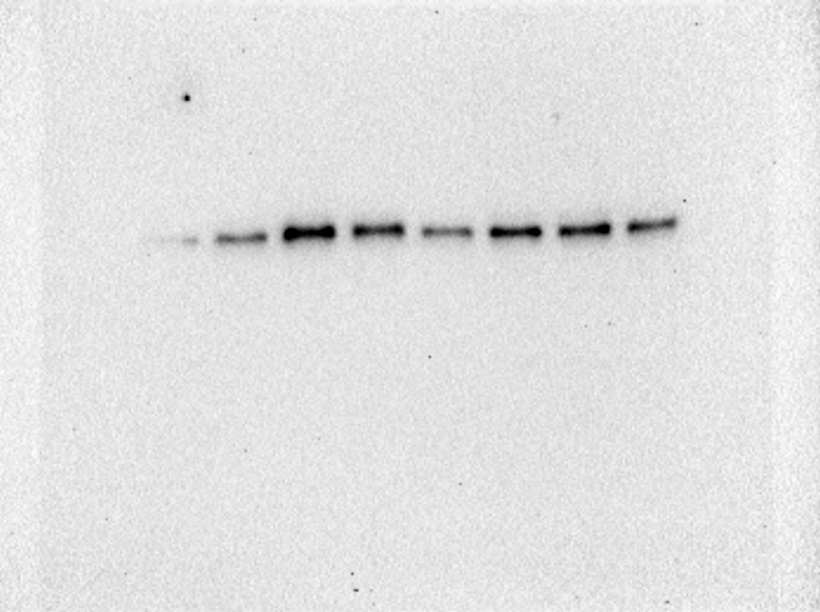


Replicate 2 of the WB for the measurement of SMAD3


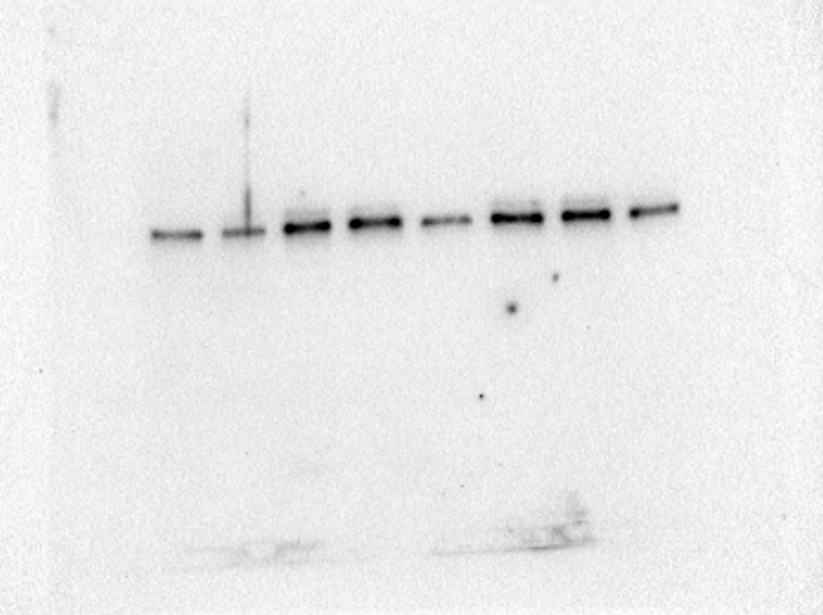


Replicate 3 of the WB for the measurement of SMAD3


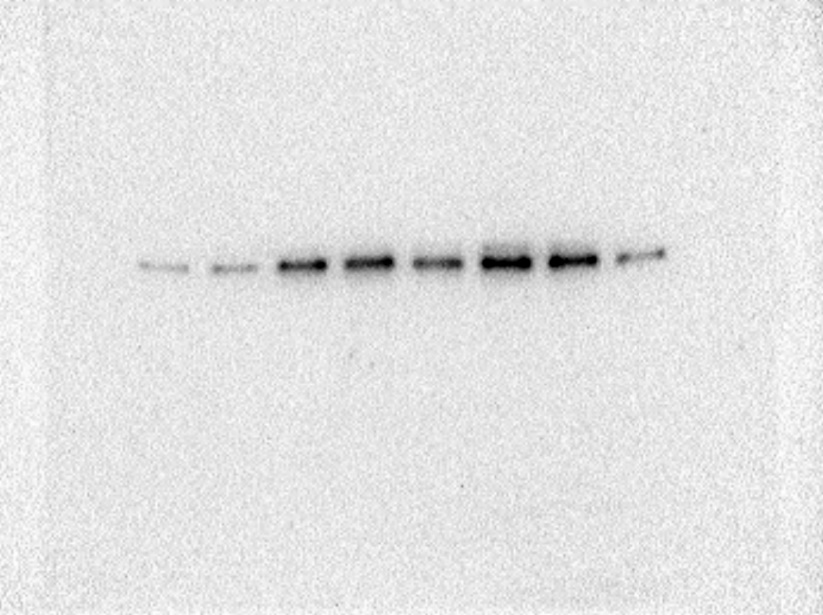


Replicate 1 of the WB for the measurement of p-SMAD2


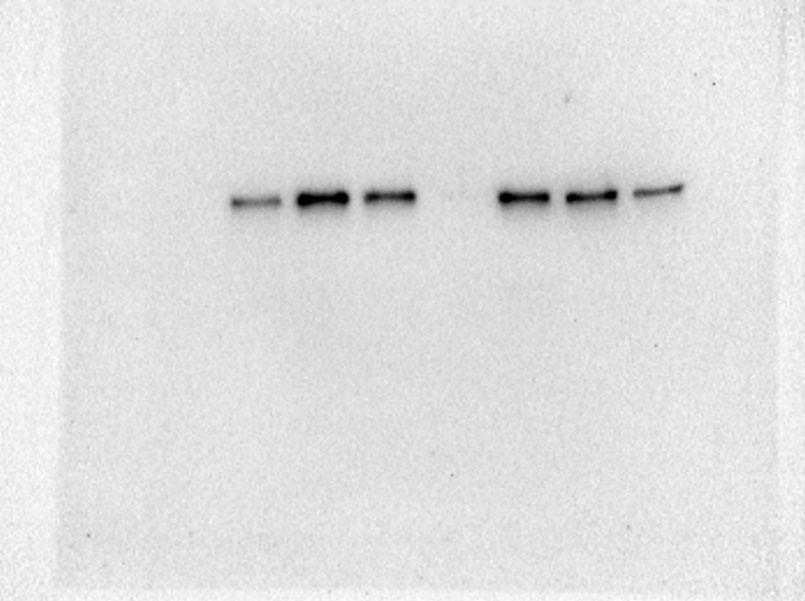


Replicate 2 of the WB for the measurement of p-SMAD2


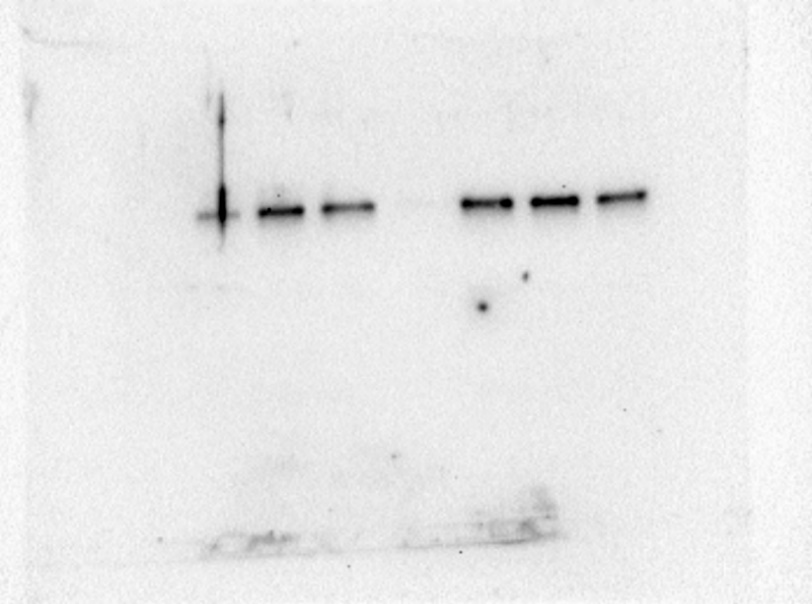


Replicate 3 of the WB for the measurement of p-SMAD2


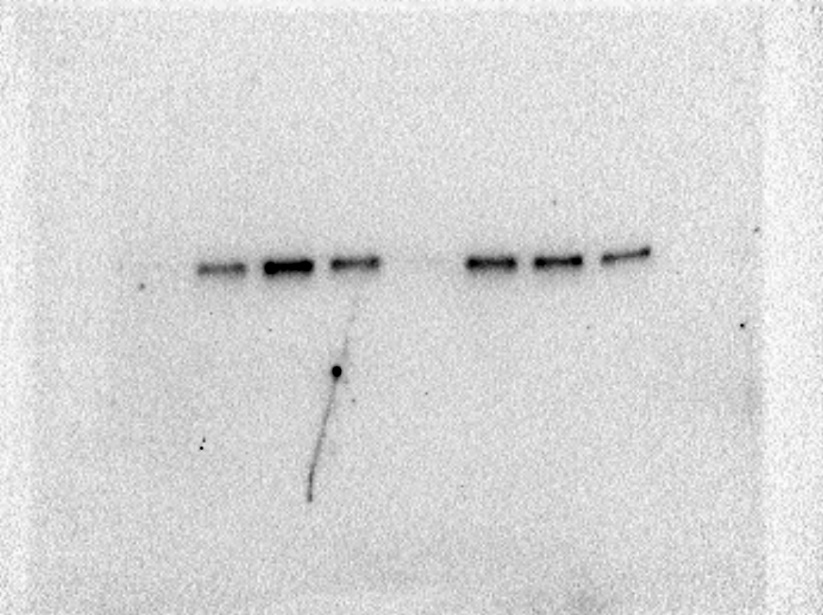


Replicate 1 of the WB for the measurement of SMAD2


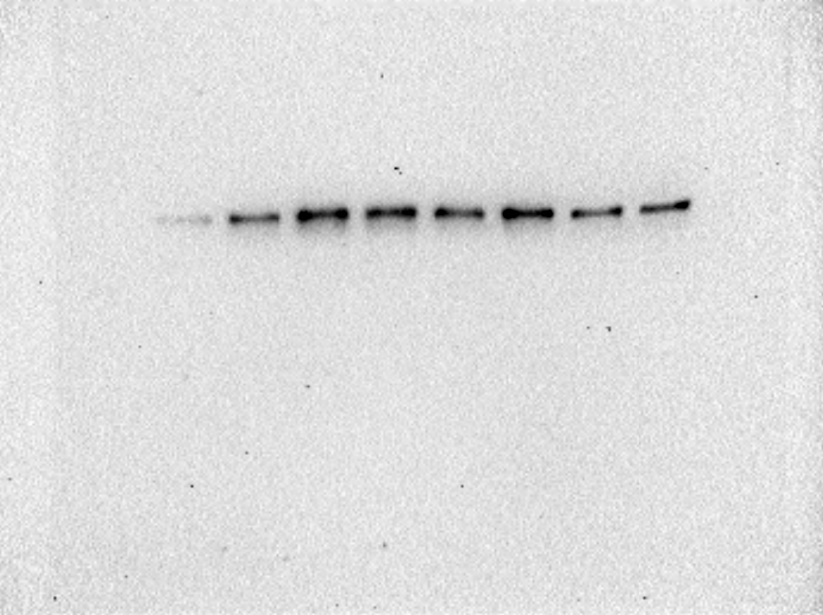


Replicate 2 of the WB for the measurement of SMAD2


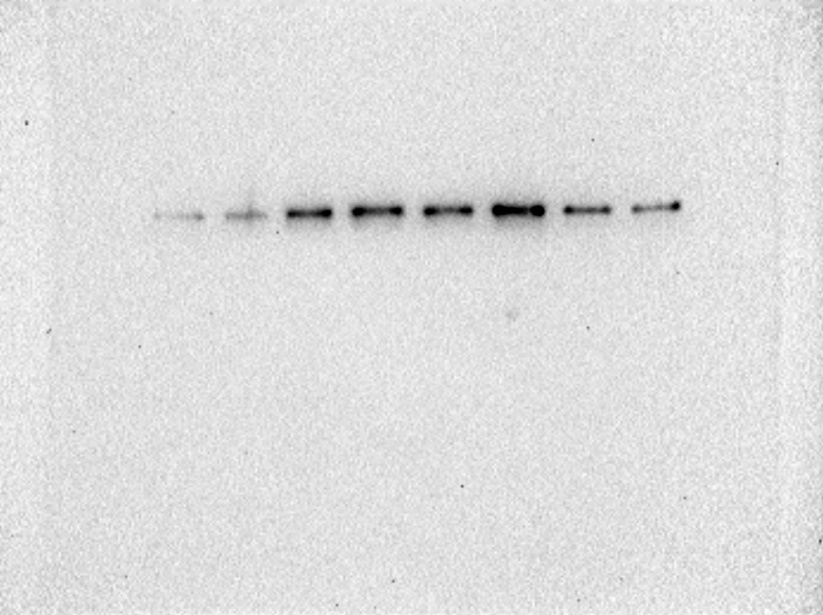


Replicate 3 of the WB for the measurement of SMAD2


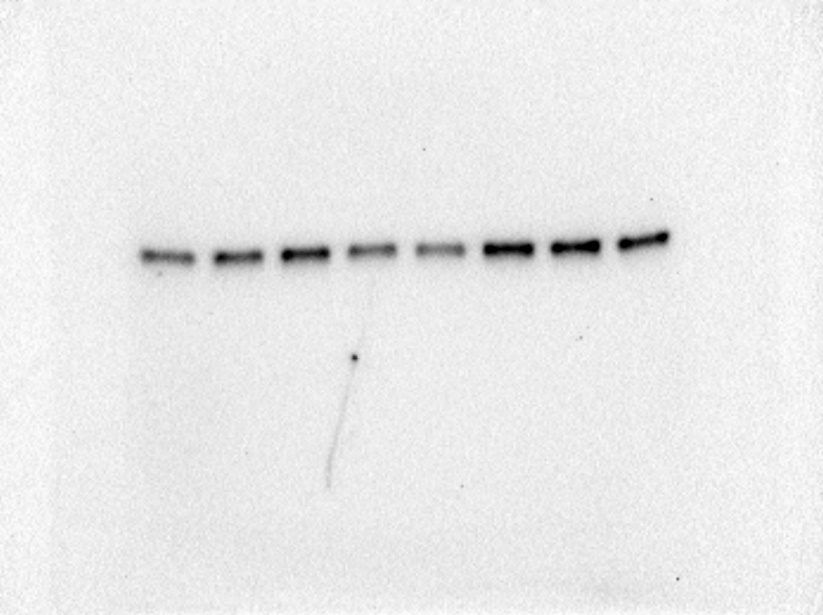


Coomassie replicate 1:


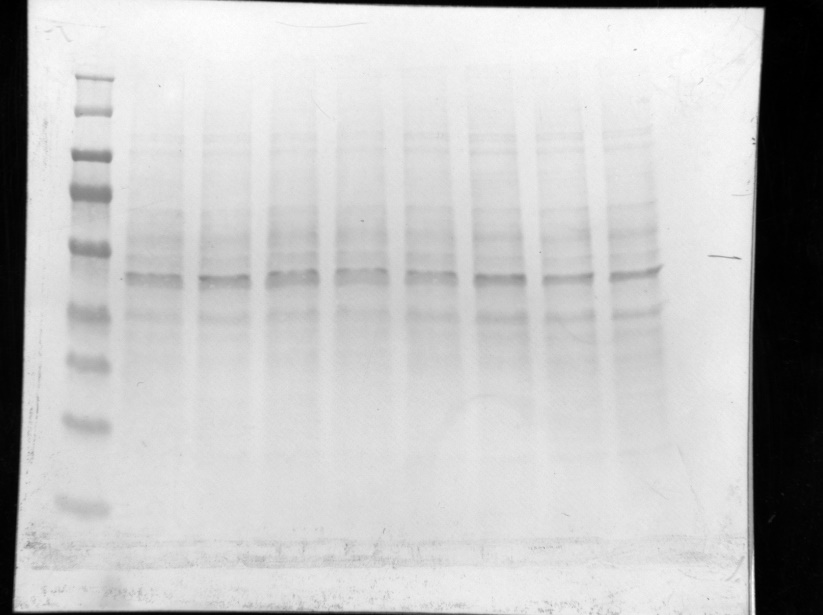


Coomassie replicate 2:


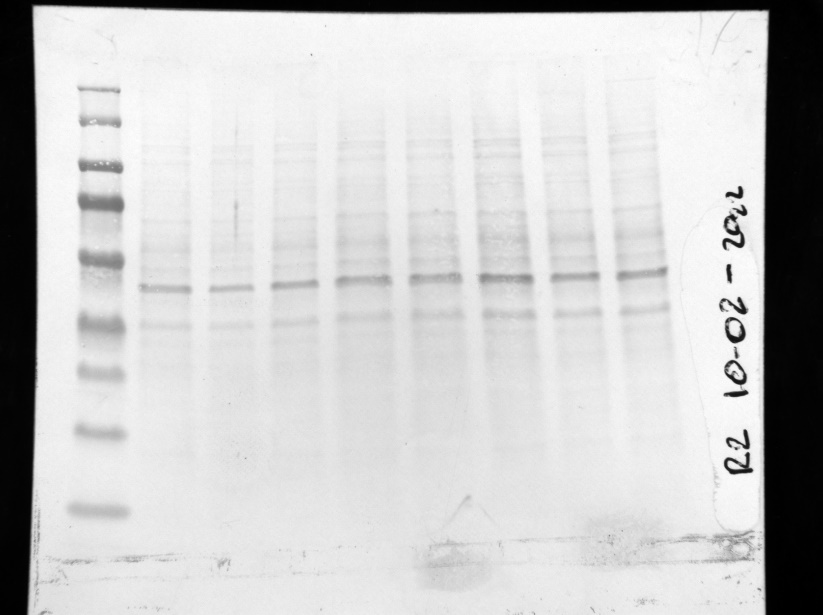


Coomassie replicate 3:


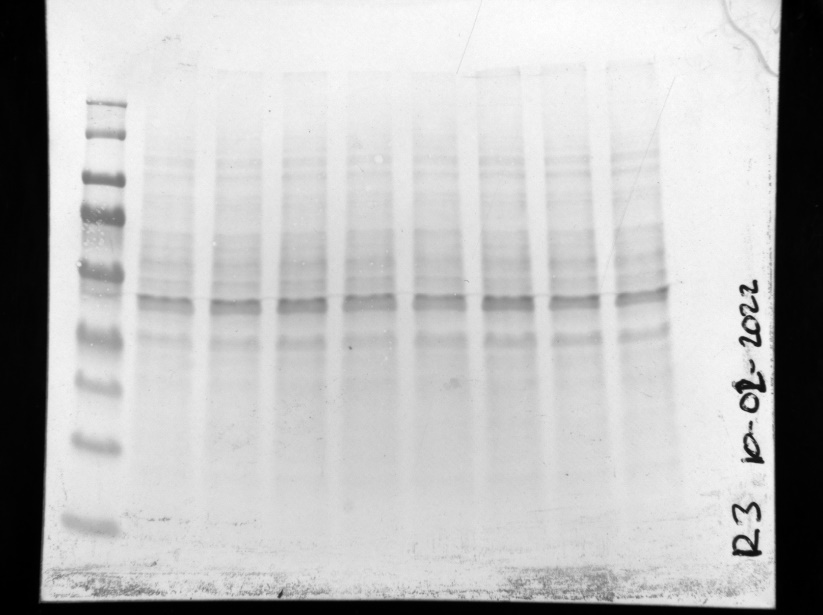


### HeLa western blots:

Replicate 1 of the WB for the measurement of p-SMAD3.


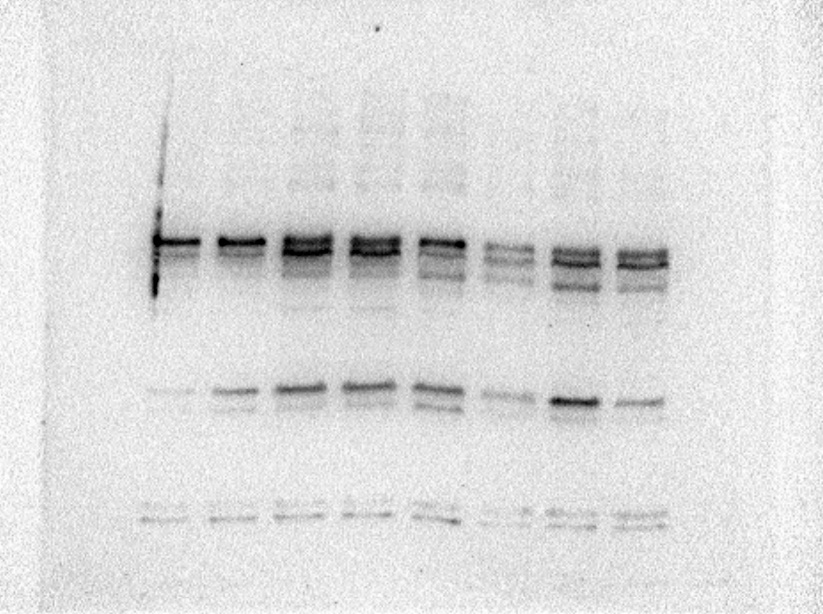


**p-SMAD3**

Replicate 2 of the WB for the measurement of p-SMAD3.


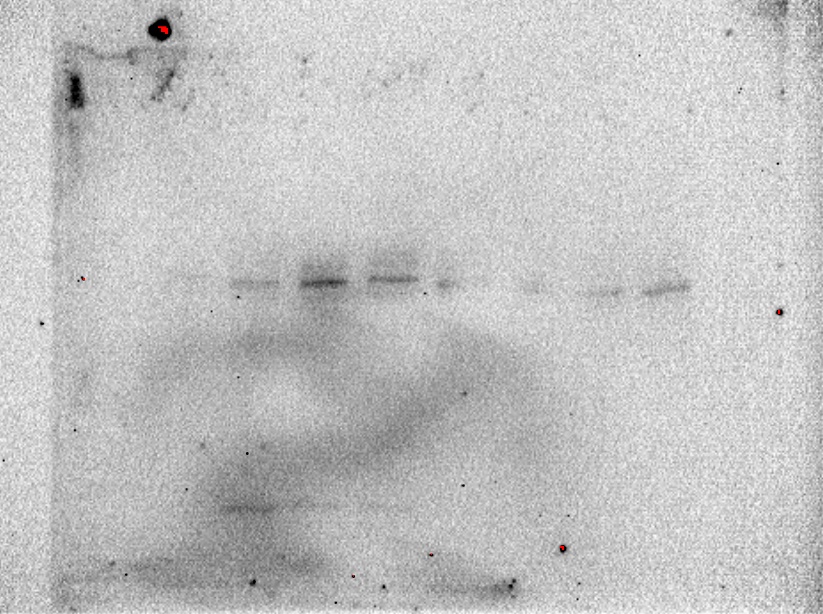


**p-SMAD3**

Replicate 1 of the WB for the measurement of p-SMAD2.


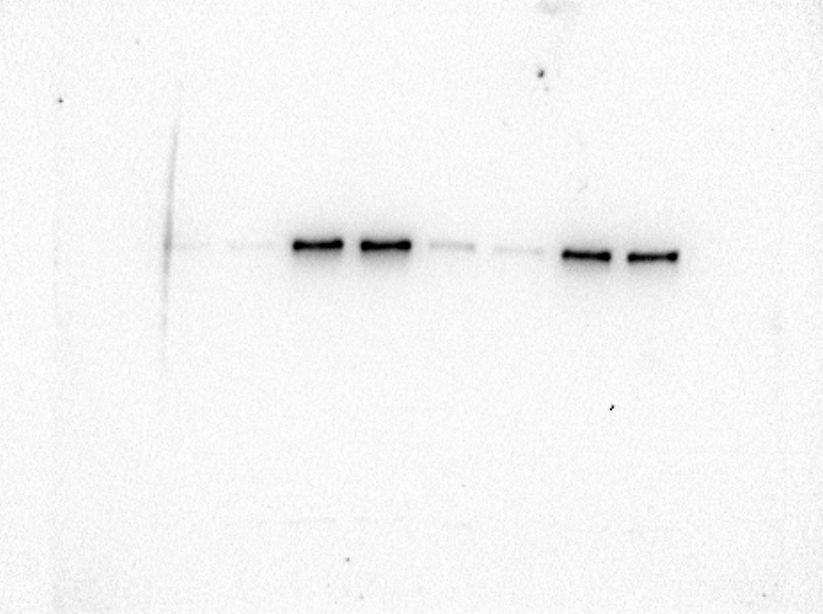


Replicate 2 of the WB for the measurement of p-SMAD2.


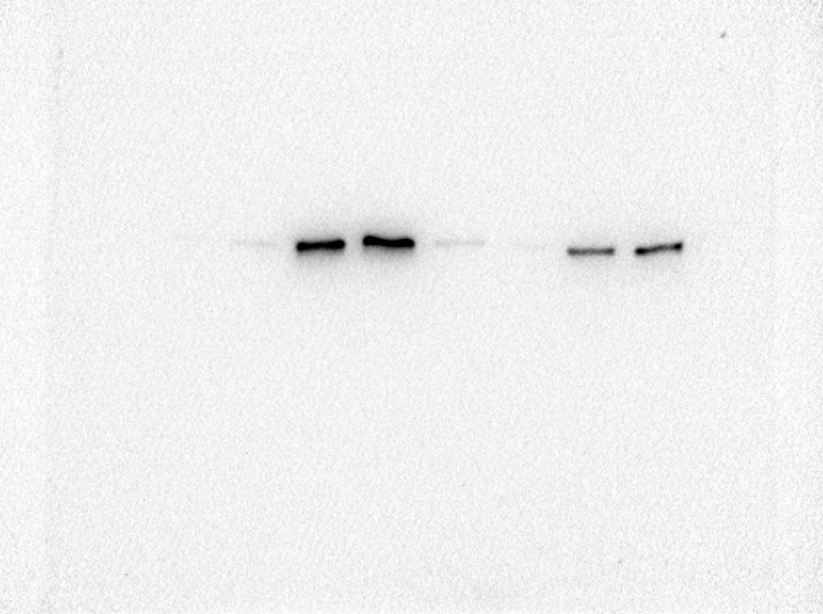


Replicate 1 of the WB for the measurement of SMAD3.


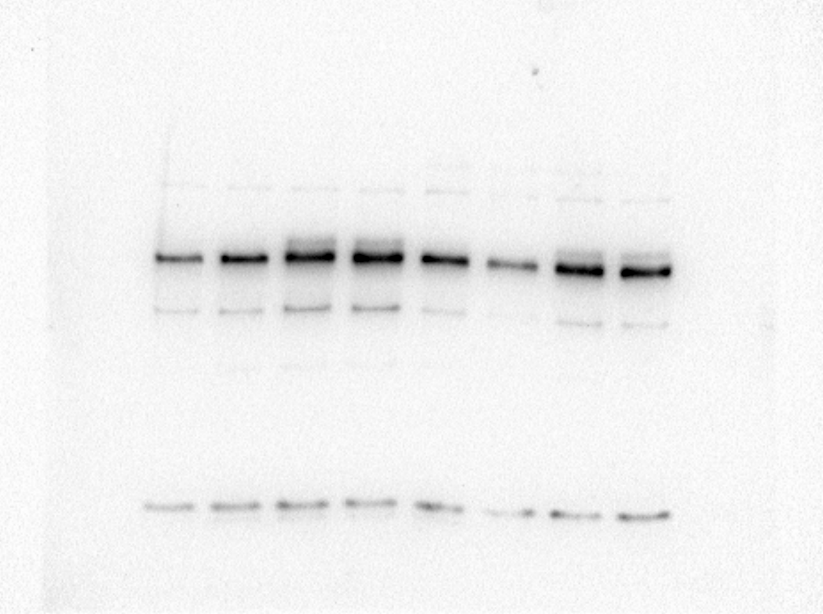


**SMAD3**

Replicate 2 of the WB for the measurement of SMAD3.


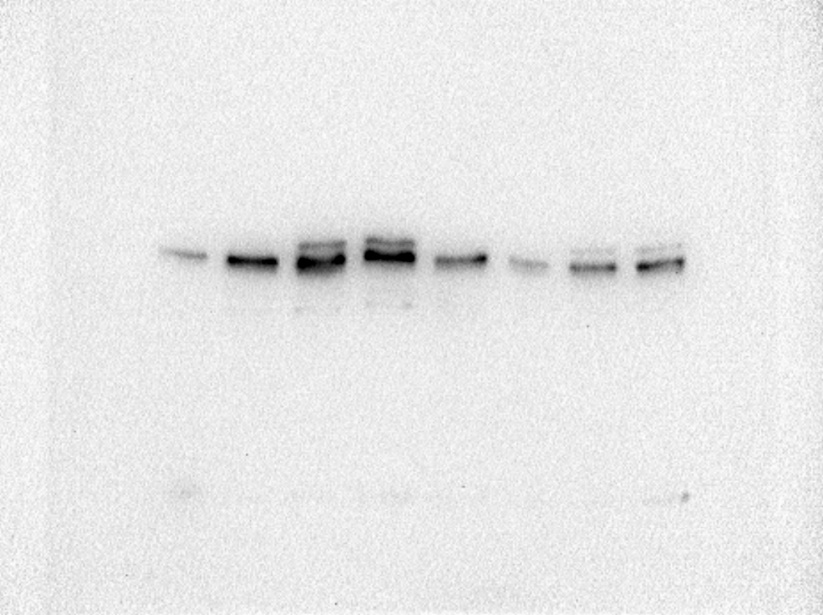


Replicate 1 of the WB for the measurement of SMAD2.


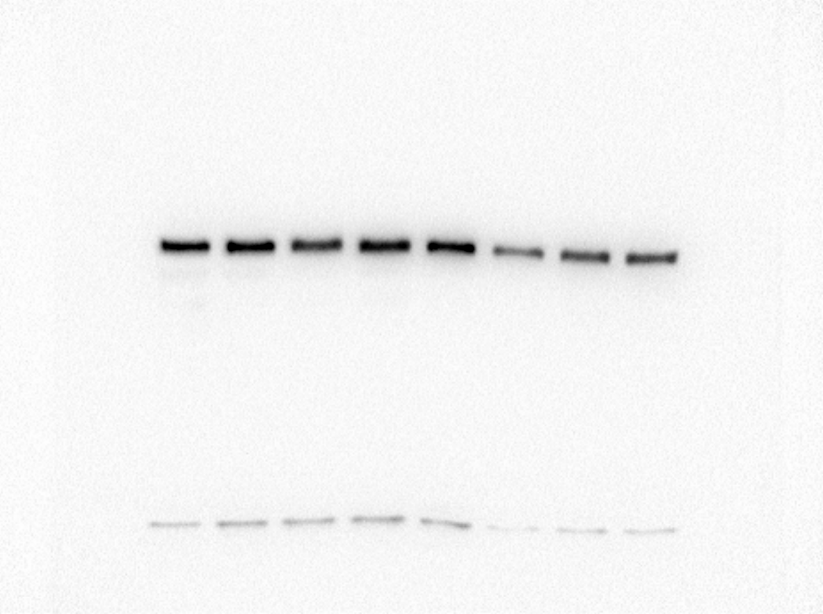


Replicate 2 of the WB for the measurement of SMAD2.


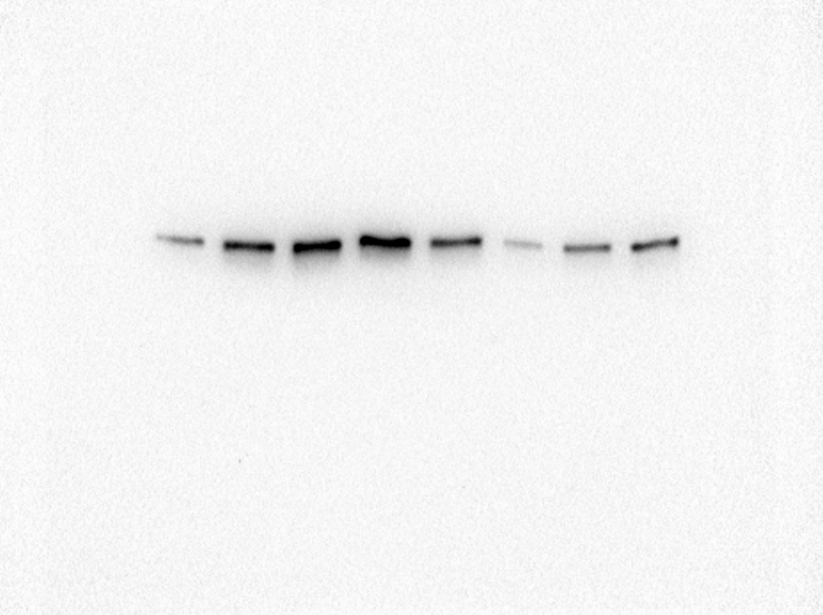


Coomassie replicate 1:


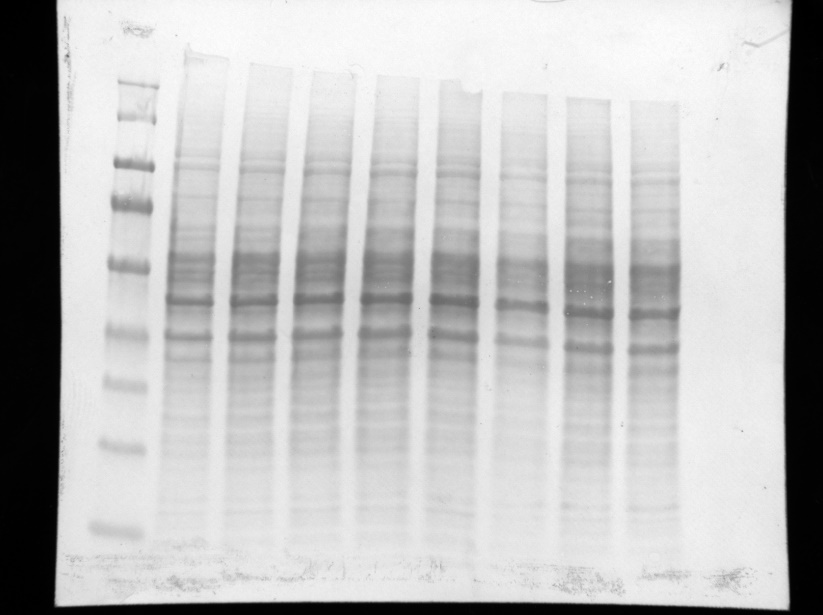


Coomassie replicate 2:


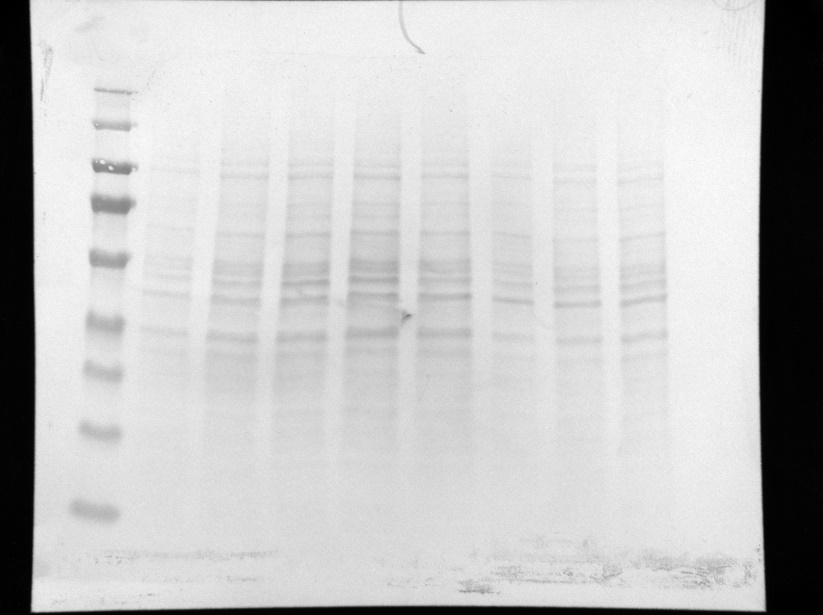

Supplement: Supplementary file 2 [file DataSheet1.docx]
